# The Characteristically Slow Proton Transfer Coupled to Platinum Oxidation in Alkaline Polyelectrolyte as Elucidated at the Molecular Level

**DOI:** 10.1021/acscentsci.5c00124

**Published:** 2025-04-30

**Authors:** Mo-Li Huang, Wenhui Ling, Zhangrui Wang, Yang Lu, Hong-Ning Shen, Li-Wen Wu, Chiyan Liu, Yong Han, Zhi Liu, Bo Yang, Yi-Fan Huang

**Affiliations:** † School of Physical Science and Technology, 387433 ShanghaiTech University, Shanghai 201210, China; ∥ State Key Laboratory of Functional Materials for Informatics, 53017 Shanghai Institute of Microsystem and Information Technology, Chinese Academy of Sciences, Shanghai 200050, China; § Center for Transformative Science, 387433 ShanghaiTech University, Shanghai 201210, China

## Abstract

The proton transfer in alkaline polyelectrolyte membrane (APEM)/electrode interfaces is significantly coupled to the electrochemical reactions in energy conversion and green synthesis. The OH^–^ in APEM/electrode interfaces is characteristically without cations in the surroundings but ambiguous in proton-transfer-coupled electrochemical reactions at the molecular level. Here we employed *in situ* electrochemical surface-enhanced Raman spectroscopy and high-level quantum-chemical calculations to elucidate the proton transfer in the APEM/Pt interface by using electrochemical Pt oxidation as an indicator. To manifest the characters in APEM, a comparison to that in conventional NaOH solution was made. With the similar electron transfer of Pt oxidation in both APEM and NaOH, the driving force and rate of proton transfer were distinguished respectively according to the onset oxidation potential and morphology of Pt nanoparticles, which suggested the slow proton transfer in an APEM/Pt interface. The similar vibrational fingerprints of subsurface oxygenated intermediates in both APEM and NaOH solution evidenced the characteristically slow proton transfer in an APEM/Pt interface. The high-level quantum-chemical calculations combined with molecular dynamics simulation showed that the driving force of proton transfer in APEM was reduced since OH^–^ was coordinated by more water molecules in its hydration shell. The characteristically slow interfacial proton transfer may be universally coupled to electrochemical reactions in devices with APEMs.

## Graphical abstract

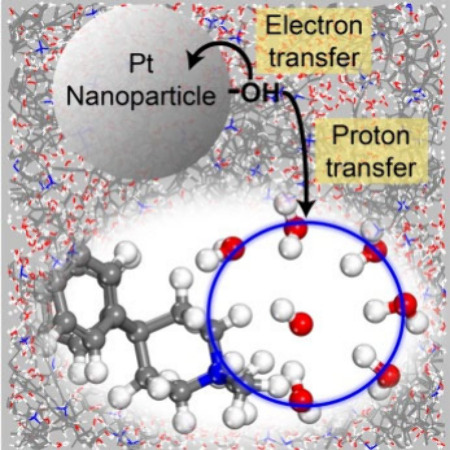

## Introduction

Alkaline polyelectrolyte membranes (APEMs) have been more and more popular in practical electrochemical devices of energy storage and green synthesis, such as water splitting, hydrogen oxidation reaction, CO_2_ reduction reaction, *etc*.^1–3^ In the interfaces between the APEM and the anode or cathode of these devices, proton transfer is accepted by the hydroxide anion (OH^–^) and coupled to electrochemical reactions. The proton transfer in APEM/electrode interfaces may drastically change the driving force and barrier of electrochemical reactions through proton-coupled electron transfer, which highly determines the overall efficiency of the devices.^4–9^ Therefore, it is pivotal and highly desired to fundamentally understand the character of proton transfer in APEM/electrode interfaces.

The proton transfer in APEM/electrode interfaces may be unique due to the structure of APEMs. In the bulk APEM, the OH^–^ is mobile, and cationic functional groups are bonded on the polymer matrix. The theoretical predictions and our recent spectroscopic characterization suggested that OH^–^ spills from the bulk APEM and migrates to the APEM/electrode interface. The spilled OH^–^ is without cations in the surroundings, which is different from that in conventional alkaline solution regarding solvation structures.^10–16^ The investigations in bulk aqueous solution have shown that proton transfer is strongly correlated to the solvation structures of ions.^3,10,17–19^ Thus, the proton transfer in the APEM/electrode interface should be different from that in conventional alkaline solution. However, the character of proton transfer in the APEM/electrode interface remains ambiguous.

The characters of proton transfer in the APEM/electrode interface may be elucidated by comparing the proton-transfer-coupled electrochemical reactions in APEM and alkaline solution, where the electron transfer is supposed to be similar. So far, the thermodynamics and kinetics of proton transfer in electrolyte/electrode interfaces were mainly inferred from electrochemical reaction equilibrium potentials and rates in solution,^7,8^ which may be quantitatively compared to those in APEM. In addition, the comparisons about surface intermediates at the molecular level will correlate proton transfer to the OH^–^ in APEM/electrode interfaces, which may be used for universally understanding the electrochemical reactions in APEMs.

Here we explored the proton transfer in the interface between an APEM of quaternary ammonia poly­(*N*-methylpiperidine-*co*-*p*-terphenyl) (QAPPT) and Pt, which was coupled to the electrochemical oxidation of the Pt surface. Proton-transfer-coupled electrochemical Pt oxidation has been an extensively studied model and is mainly composed of the elementary steps delineated by eqs 1 and 2:^20,21^

1 $$\mathrm{Pt}+nH_{2}O⇄\mathrm{Pt}{\left(\mathrm{OH}\right)}_{n}+nH^{+}+ne^{-}$$

2 $$H^{+}+{\mathrm{OH}}^{-}⇄H_{2}O$$

In this way, the thermodynamic driving forces of proton transfer in APEM and alkaline solution were quantitatively compared according to the Pt oxidation potential. The kinetics of proton transfer was quantitatively examined according to the morphological changes of monodispersed Pt nanoparticles due to the accumulation of surface hydroxygenated species with irreversible repeated oxidation and reduction cycles.^22–25^ The surface intermediates of proton-transfer-coupled electrochemical Pt oxidation were characterized to compare the reaction mechanisms in APEM and alkaline solution by using *in situ* electrochemical surface-enhanced Raman spectroscopy (SERS).^26^ Finally, the characteristic proton transfer in the QAPPT/Pt interface was correlated to OH^–^ according to the structures as simulated by molecular dynamics and the driving force of proton transfer as calculated by high-level quantum-chemical methods.

## Results and Discussion

### Driving Forces and Rates of Proton-Transfer-Coupled Electrochemical Pt Oxidation in (Poly)­electrolyte/Pt Interfaces

To manifest morphological changes, monodispersed Pt nanoparticles were employed to demonstrate proton transfer coupled to electrochemical Pt oxidation. As shown by the scanning electron microscopy (SEM) image in Figure 1a, the monodispersed Pt nanoparticles (275 ± 1 nm) were electrochemically deposited on a glassy carbon electrode. With a three-electrode system, the voltammograms of these monodispersed Pt nanoparticles were measured in QAPPT and a 0.1 M NaOH solution. As shown in Figure 1b, the features of these voltammograms indicated that the surface of monodispersed Pt nanoparticles was polycrystalline. The onset oxidation potential in QAPPT (0.77 V) was 80 mV more positive than that in the NaOH solution (0.69 V). To obtain a perfect voltammogram, the *iR* drop from the resistance of a polyelectrolyte membrane should be compensated well. Because the onset oxidation potential was identified with a very tiny current, the influence from the resistance of QAPPT on the identification of an onset potential was negligible in our voltammograms without *iR* drop compensation.

**Figure 1:**
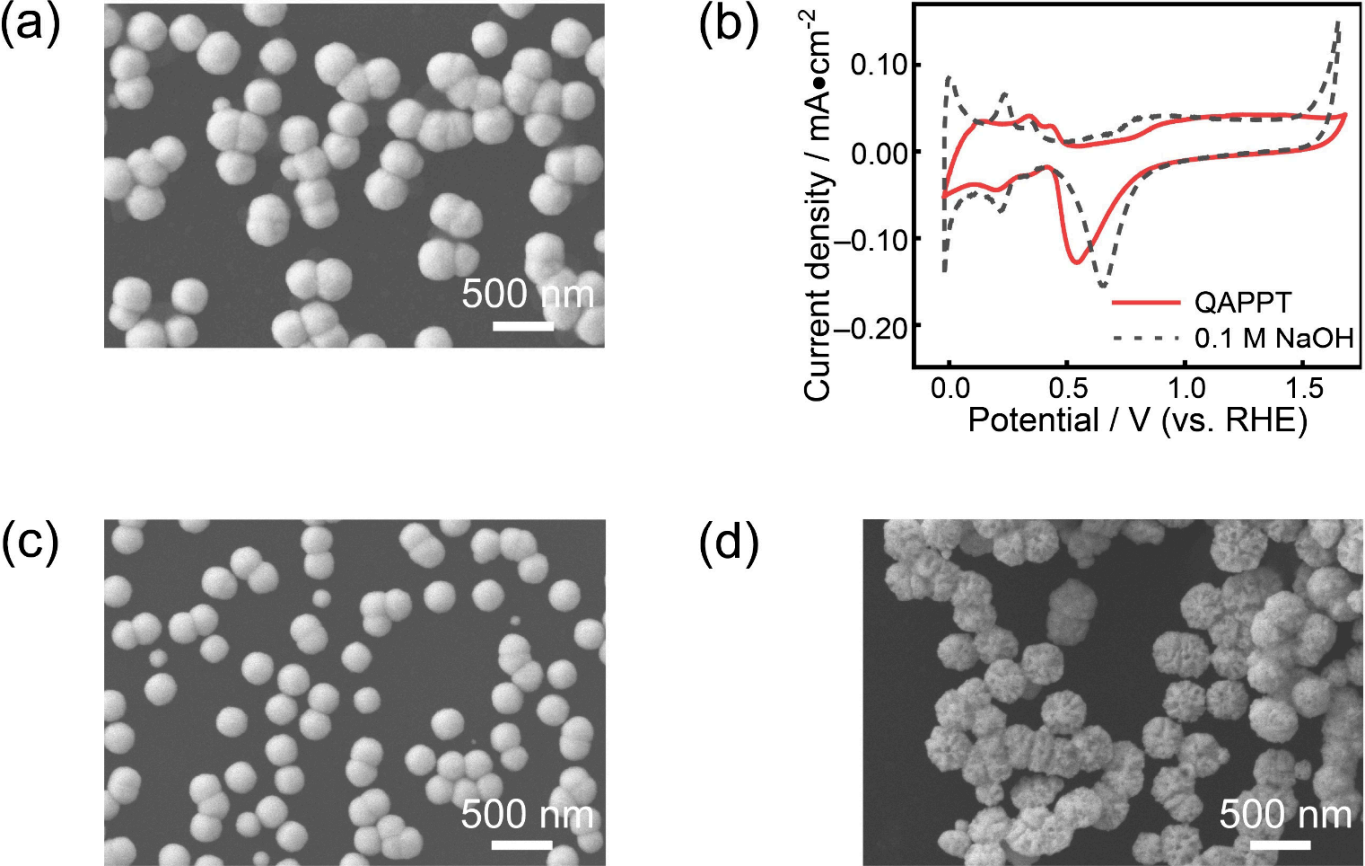
(a) SEM image of monodispersed Pt nanoparticles on a glassy carbon electrode. (b) Voltammograms of monodispersed Pt nanoparticles in QAPPT (solid line) and 0.1 M NaOH solution (dashed line) with a scan rate of 50 mV/s. (c, d) SEM images of monodispersed Pt nanoparticles with square potential cycles of oxidation at 1.5 V and reduction at 0.2 V for 1 h in (c) QAPPT and (d) 0.1 M NaOH.

Usually, the onset potential of Pt oxidation may be complicatedly influenced by the adsorption of ions (such as sulfate), the types of nonadsorbed electrolyte, and the equilibrium shifts of Pt oxidation as demonstrated by eqs 1 and 2.^27,28^ The more positive onset potential of Pt oxidation indicated that either the thermodynamic driving force of proton transfer in QAPPT is smaller than that in NaOH solution or QAPPT is chemically adsorbed on the surface.

The morphological changes of monodispersed Pt nanoparticles with repeated irreversible oxidation–reduction cycles were used to examine the kinetics of interfacial proton transfer in QAPPT and NaOH solutions. The surface of the Pt nanoparticles was completely oxidized and reduced through multiple cycles during prolonged treatment. Figure 1c,d shows SEM images with the oxidation (1.5 V)–reduction (0.2 V) cycles for 1 h in QAPPT and NaOH solution, respectively. In QAPPT, the morphology of Pt nanoparticles was similar to that as prepared (Figure 1a). In contrast, roughness appeared on each Pt nanoparticle in NaOH solution. Because metallic Pt is stable at room temperature, the roughness on Pt nanoparticles should be due to the dissolution–reduction or surface migration of Pt hydroxides.^22–25^ The smaller morphological changes of Pt nanoparticles in QAPPT were therefore ascribed to either surface hydroxygenated species with lower coverage from slow proton transfer or inhibited migration of Pt hydroxides.

### Slow Proton Transfer in an APEM/Pt Interface as Supported by the Mechanisms of Electrochemical Pt Oxidation

Considering that the driving forces and rates of proton-transfer-coupled electrochemical Pt oxidation and Pt hydroxide migration might be changed once the ionic functional groups in the PEM are chemically adsorbed,^29,30^ we compared the surface intermediates of electrochemical Pt oxidation in QAPPT and NaOH solution to support the slow interfacial proton transfer in QAPPT at the molecular level. *In situ* electrochemical SERS measurements were performed to diagnose surface intermediates by using our recently reported homemade spectroelectrochemical cell with a three-electrode system.^26^ As illustrated by Figure 2a, the SERS-active Au-core@Pt-shell nanoparticles were loaded with a transparent carbon film, which acted as a working electrode. With a layer-by-layer structure, a quartz window, a Au mesh, a QAPPT film, and a Pt counter electrode were mechanically stacked and sealed in a closed cell. The SERS signal of the QAPPT/Pt interface was excited and collected through the quartz and carbon film. Figure 2b shows the potential-dependent SERS spectra of the QAPPT/Pt interface with an initial potential of 0.6 V and positively stepped potentials. A broad band at 400 cm^–1^ was the background of the spectroelectrochemical cell. At 1.2 V, a band at 562 cm^–1^ appeared, whose frequency shifted to 580 cm^–1^ at 1.6 V and whose intensity steadily increased with respect to the positively shifted potential. A similar band was also observed in a 0.1 M NaOH solution, as shown in Figure 2c. This band at 562 cm^–1^ has been assigned to the Pt–O stretching modes (ν_Pt–O_) of amorphous Pt hydroxides,^31–33^ and therefore, the electrochemical Pt oxidation in both QAPPT and NaOH solution had the same intermediates and proceeded by similar mechanisms. There was not any band related to chemically adsorbed QAPPT, and thus, the interaction between the Pt surface (metallic Pt or Pt hydroxides) and QAPPT was weak. The inhibitory effect of QAPPT on the morphological changes of Pt nanoparticles was neglected (see the Supporting Information for a discussion about the interaction between Pt and QAPPT). Therefore, the onset Pt oxidation potential shift and morphological changes of Pt nanoparticles were attributed to the proton-transfer-coupled production of surface intermediates.

**Figure 2:**
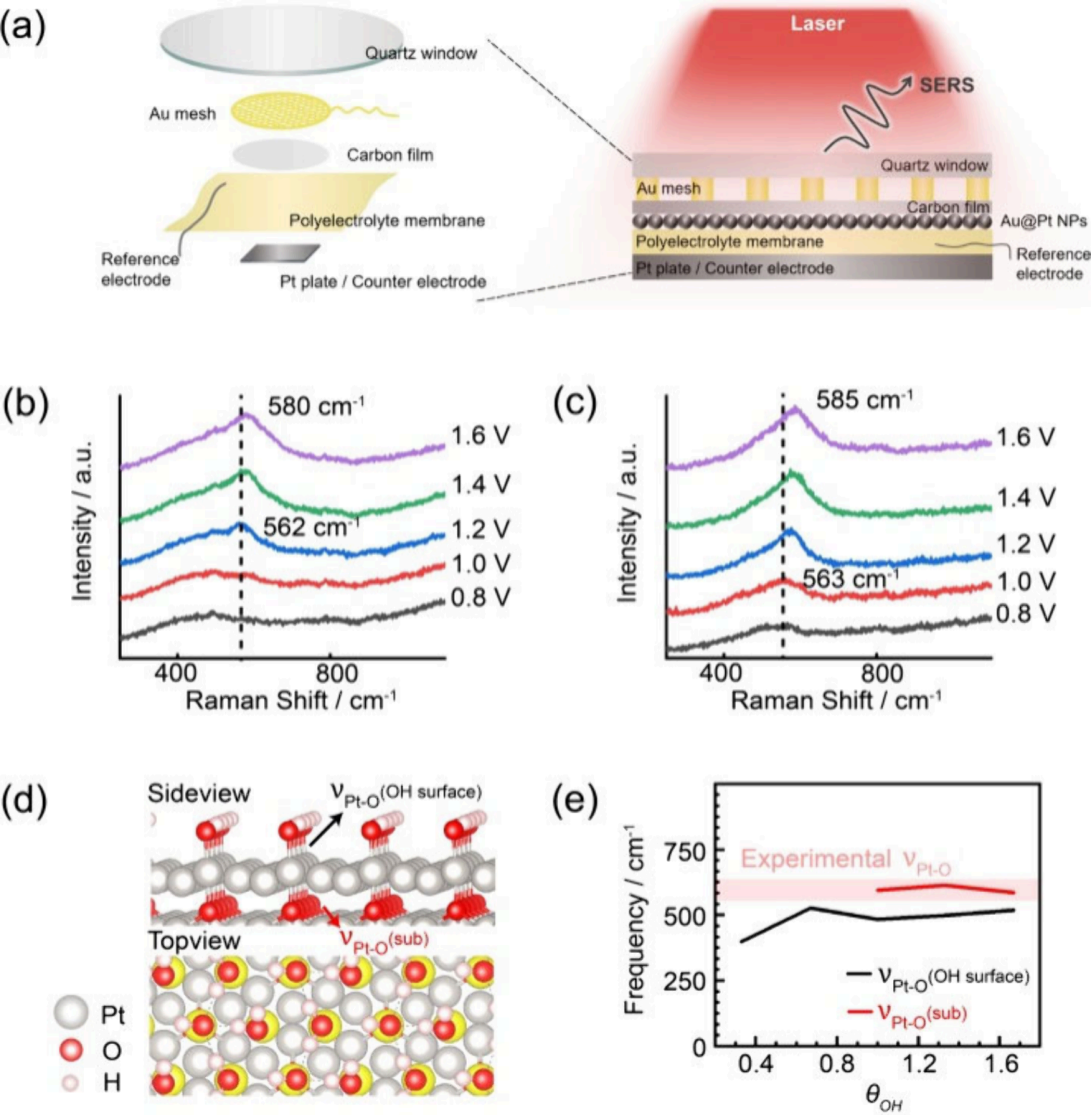
(a) Schematic illustration of the *in situ* EC-SERS in polyelectrolyte. (b, c) Potential-dependent SERS spectra of electrochemical Pt oxidation in (b) QAPPT and (c) 0.1 M NaOH solution. (d) Side view (the solvated H_2_O on the surface is not shown) and top view of Pt hydroxides. The highlighted atoms are in the second layer of the Pt(111) slab. (e) Calculated ν_Pt–O_ of Pt hydroxides with a series of coverage of surface oxygenated species (θ_OH_).

Notably, the band of ν_Pt–O_ at 563 cm^–1^ appeared at 1.2 V in QAPPT, which was more positive than that at 1.0 V in the NaOH solution. This more positive onset potential of ν_Pt–O_ provided molecular evidence of the slow proton transfer in QAPPT. To demonstrate this evidence, we employed density functional theory to reproduce the vibrational frequencies of ν_Pt–O_ on Pt(111), Pt(553), and Pt(533) slabs as a function of the coverage of surface hydroxygenated species. For instance, as shown in Figure 2e, the ν_Pt–O_ in the subsurface and on the surface of Pt(111) gave the frequencies of 594 and 483 cm^–1^, which suggested that the band at 563 cm^–1^ in the experimental SERS spectra was assigned to subsurface oxygenated species. With the coverage of surface hydroxygenated species more than 2/3 as shown in Figure 2e, the subsurface oxygenated species were produced and ν_Pt–O_ was blue-shifted, which reproduced well the potential-dependent SERS spectra. Previous studies have attributed the production of subsurface oxygenated species to the accumulation of surface hydroxygenated species through a place-exchange process.^20^ The subsurface oxygenated species are key intermediates in the surface roughness evolution. Therefore, the more positive onset potential of ν_Pt–O_ in QAPPT indicated that the production (eqs 1 and 2) or the dehydration (2Pt_surf_OH ⇄ Pt_surf_O_subsurf_ + Pt_surf_(H_2_O) of surface hydrogenated species is slow, which suggests slow proton transfer.

### Correlating the Slow Proton Transfer in APEM/Pt Interfaces to the Hydration Structure of OH^–^ Spilled from the APEM

To demonstrate that slow proton transfer is universal in the electrochemical reactions in APEM, we employed theoretical methods to correlate the slow proton transfer in QAPPT/Pt interfaces to the hydration structure of OH^–^ in terms of the driving force of proton transfer. Molecular dynamics simulations were performed to compare the local structures of OH^–^ in QAPPT and NaOH solution. Because the hydration of OH^–^ in solution is dynamic at room temperature, an averaged result at equilibrium was necessary for understanding the local structures of OH^–^. Figure 3a shows the radial distribution function (*g*(*r*)) of H_2_O as a function of the distance to OH^–^ at equilibrium with long time simulation. The sharp peaks at a distance of 2.7 Å corresponded to the water molecules coordinated to OH^–^. The coordination number (red dashed lines in Figure 3a) in QAPPT was approximately 6.3, which is larger than that of 5.6 in NaOH. The typical structures of the OH^–^ coordinated by water molecules in QAPPT and NaOH are demonstrated by Figure 3b,c, respectively. The OH^–^ with more coordinated H_2_O in QAPPT was consistent with our previous estimation of larger interionic distance regarding the Debye length.^16^ Generally, in the QAPPT/Pt interface, there are no cations in the surroundings of the spilled OH^–^. More H_2_O molecules are coordinated to OH^–^ in order to reduce interionic repulsion, where H_2_O molecules are rotatable dipoles. Hence, the different local structures of OH^–^ in QAPPT and NaOH solution hint that the coordinated H_2_O number of OH^–^ is a key clue for understanding the slow proton transfer in QAPPT.

**Figure 3:**
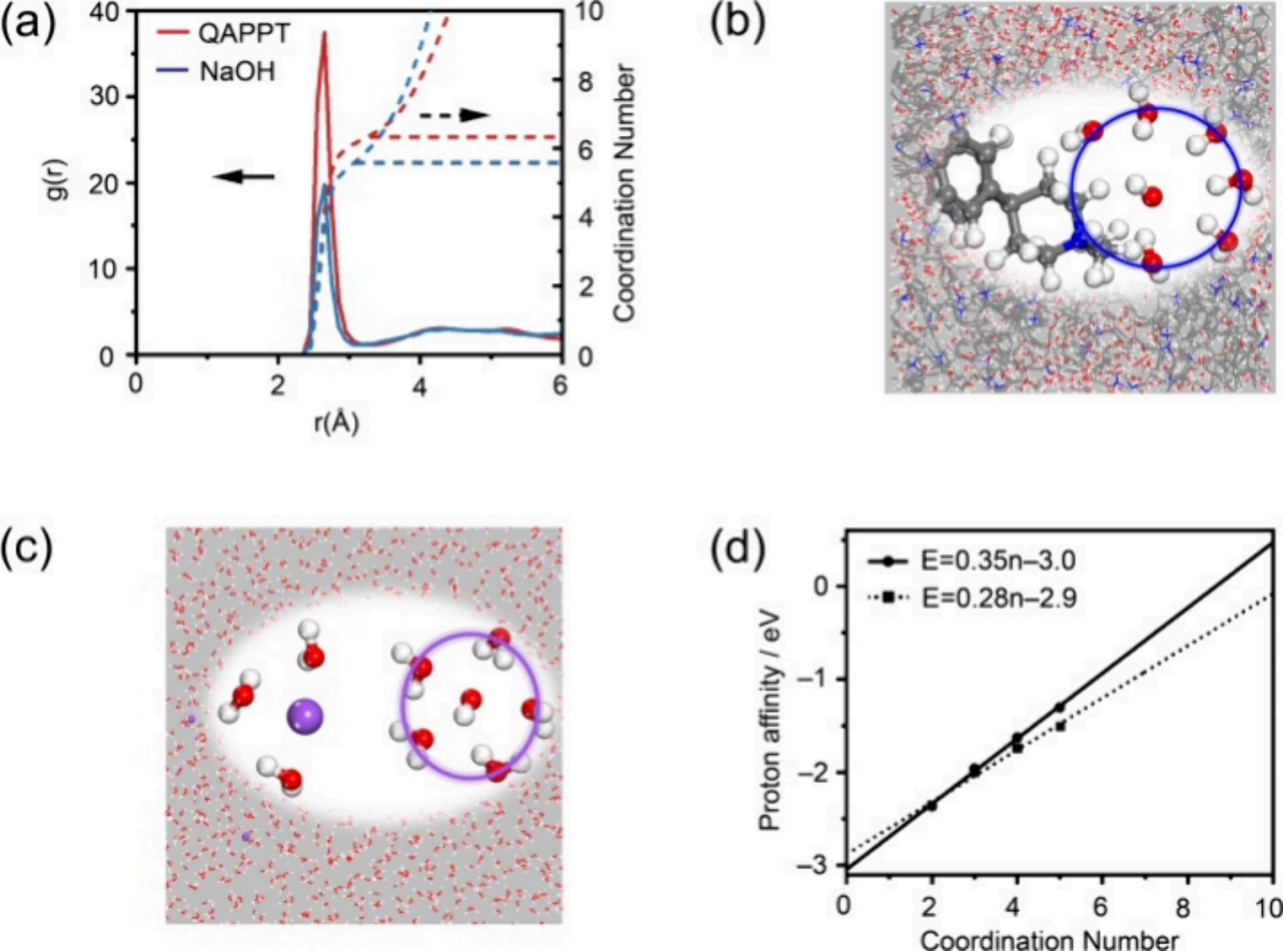
(a) Molecular-dynamics-simulated radial distribution function of H_2_O as a function of the distance to OH^–^. (b, c) Typical structures of the hydrated OH^–^ in (b) QAPPT and (c) 0.1 M NaOH solution. (d) Quantum-chemistry-calculated proton affinity of OH^–^(H_2_O)_*n*_ (dashed line, MP2-aug-cc-PVTZ; solid line, CCSD/aug-cc-PVQZ correction).

The proton affinity of OH^–^ as a function of the number of coordinated H_2_O, which was obtained by a high-level quantum-chemical calculation, was used to demonstrate the slow proton transfer. Recent experimental and theoretical studies showed that the properties of OH^–^ in solution were reproduced well by the clusters of OH^–^(H_2_O)_*n*_.^10,34,35^ Based on these OH^–^(H_2_O)_*n*_ clusters, we calculated their proton affinity according to the reaction of H_3_O^+^ + OH^–^(H_2_O)_*n*_ ⇄ (*n* + 2)­H_2_O. The coupled-cluster method (CCSD) with the aug-cc-PVQZ basis set was employed to improve the calculation accuracy of proton affinity on the geometries optimized by using the MP2 method with the aug-cc-PVTZ basis set. As summarized in Figure 3d, with the increase in the number of coordinated H_2_O, the proton affinity of OH^–^(H_2_O)_*n*_ decreased monotonically. In other words, OH^–^ with more coordinated H_2_O accepted a proton with more difficulty. The proton affinity of OH^–^(H_2_O)_6.3_ (QAPPT) was estimated to be 245 meV lower than that of OH^–^(H_2_O)_5.6_ (NaOH) according to the linearly fitted function at the CCSD/aug-cc-PVQZ level. These results were also consistent with the simulation results that the hypercoordination disfavored proton transfer in bulk solution.^19^ In addition to a thermodynamic driving force, the barrier of proton transfer was more straightforward for understanding the kinetics. Unfortunately, it remains a challenge to estimate the barriers of proton transfer and proton-transfer-coupled electrochemical reactions in interfaces. Phenomenologically, the barrier of proton transfer is contributed by the reorganization energy of the surrounding of surface hydroxygenated species.^6,36–38^ Further measurements and simulations about the hydration shell of surface hydroxygenated species and the dielectric constant in the electric double layer of the QAPPT/Pt interface would help with quantitatively understanding the kinetics of proton transfer.

Although the OH^–^(H_2_O)_*n*_ clusters are not completely equal to that in APEM/electrode interfaces, it can be expected that the slow proton transfer in QAPPT arises essentially because the spilled OH^–^ on the electrode surface have more coordinated H_2_O and lower proton affinity in a local hydration shell. Since the transferred proton is accepted by the OH^–^ spilled from APEM, the characteristically slow proton transfer should be universal in the APEM/electrode interfaces, and it could be reasonable to expect more characteristic proton-transfer-coupled electrochemical reactivity.

## Conclusions

As highlighted, the proton-transfer-coupled electrochemical Pt oxidation in alkaline polyelectrolyte membrane/electrode interfaces was studied. The characteristically slow proton transfer in QAPPT was found according to the more positive onset potential shift of 80 mV of Pt oxidation and smaller morphological changes of Pt nanoparticles with oxidation–reduction cycles compared to those in NaOH solution. The slow proton transfer in APEM was evidenced by the similar intermediates of subsurface oxygenated species and the lower accumulation of surface hydroxygenated species. Molecular dynamics simulations showed that the H_2_O coordination number of OH^–^ in APEM was more than that in NaOH solution. The high-level quantum-chemical calculations of the proton affinity of OH^–^(H_2_O)_*n*_ clusters indicated that the proton-transfer driving force was decreased when th OH^–^ had more coordinated water. These results essentially elucidated that the proton transfer in the QAPPT/Pt interface is characteristically slow due to the interfacial OH^–^ with more coordinated water, which may universally influence the electrochemical reactions in APEM/electrode interfaces.

## Supplementary Material



## Data Availability

Correspondence and requests for materials should be addressed to Z.L., B.Y., and Y.-F.H.

## Notes

Chemicals and materials, equipment, preparation and characterization of Au-core@Pt-shell nanoparticles, *in situ* electrochemical Raman measurements with a three-electrode cell, and molecular dynamics and quantum-chemical simulations (PDF)
