# Supplementary material for: The Characteristically Slow Proton Transfer Coupled to Platinum Oxidation in Alkaline Polyelectrolyte as Elucidated at the Molecular Level
Source: ACS Cent Sci. 2025 Apr 30;11(5):791–6. doi: 10.1021/acscentsci.5c00124 (PMC12123459; doi:10.1021/acscentsci.5c00124)
Supplement: Supplementary file 1 [file oc5c00124_si_001.pdf]

Supporting Information

The Characteristically Slow Proton-Transfer Coupled to Platinum Oxidation in Alkaline Polyelectrolyte as Elucidated at Molecular Level

Mo-Li Huang[a],‡, Wenhui Ling[a],‡, Zhangrui Wang[a], Yang Lu[a], Hong-Ning Shen[a], Li-Wen Wu[a], Chiyan Liu[b], Yong Han[a],[c], Zhi Liu[a],[c],*, Bo Yang[a],*, and Yi-Fan Huang [a],*

[a] School of Physical Science and Technology, ShanghaiTech University, Shanghai 201210, China

[b] State Key Laboratory of Functional Materials for Informatics, Shanghai Institute of Microsystem and Information Technology, Chinese Academy of Sciences, Shanghai 200050, China

[c] Center for Transformative Science, ShanghaiTech University, Shanghai 201210, China

Corresponding Author E-mail: liuzhi@shanghaitech.edu.cn; yangbo1@shanghaitech.edu.cn; huangyf@shanghaitech.edu.cn

‡Mo-Li Huang and Wenhui Ling contributed equally.

S1 Chemicals:

$HAuCl_4 \cdot 3H_2O$ was purchased from Aladdin. Sodium citrate and $H_2PtCl_6 \cdot 6H_2O$ were purchased from Sinopharm Chemical Reagent Co., Ltd. Sodium hydroxide and Perchlorate acid were purchased from Sigma-Aldrich. Ultrapure water (18.2 MΩ•cm at 300 K) was prepared by using Milli-Q system, which was used to make all aqueous solutions. Nafion (N115) and QAPPT membranes were purchased from FuelCellStore and EVE Energy Co., Ltd, respectively.

S2 Synthesis of monodispersed Pt nanoparticles and Au-core@Pt-shell nanoparticles:

The monodispersed Pt nanoparticles were electrochemically deposited on a glassy carbon electrode according to the method in a literature.[1] A glassy carbon electrode was mechanically polished and cleaned, and then transferred into an electrochemical cell. A potential step from 0.4 V to -0.4 V (vs. a saturated calomel electrode) was applied on the cleaned glassy carbon electrode and the potential of -0.4 V was held for 20 ms in 2 mM K_2PtCl_4 + 0.5 M H_2SO_4. Then an AC square potential wave between 0.6 V and 0.2 V with a frequency of 100 Hz was applied for 1h. Finally, monodispersed Pt nanoparticles were deposited on this glassy carbon electrode.

The SERS-active Au-core@Pt-shell nanoparticles were synthesized by reducing Pt salts on Au nanoparticles.[2] First, 55 nm Au nanoparticles were synthesized by quickly adding 0.35 ml 1 wt% sodium citrate aqueous solution into the boiling 50 ml 0.01 wt% $HAuCl_4$ aqueous solution under vigorously stirring. Second, 15 mL the prepared Au sol was mixed with 0.735 ml 1mM H_2PtCl_6 and 8.895 ml H_2O, once the sol was cooled to room temperature. The mixture was heated to 80 °C. Then, 0.37 ml 10 mM the ascorbic acid solution was ejected drop-by-drop with a rate of 2.25 ml/h. Finally, the solution was stirred for 30 min for completely reducing H_2PtCl_6, and Au-core@Pt-shell nanoparticles were obtained. The voltammogram in 0.1 M $HClO_4$ confirmed that the prepared Au-core@Pt-shell nanoparticles were pinhole free.[3]

S3 In-situ electrochemical surface-enhanced Raman spectroscopic measurements:

The in-situ electrochemical SERS measurements were carried out in our home-made three-electrode spectroelectrochemical cell.[4] The Ag/AgCl and Ag/Ag_2O electrodes were used as the reference electrode in Nafion and QAPPT polyelectrolyte. All the applied potentials were calculated to be with respect to a reversible hydrogen electrode according to Nernst's equation.

S4 The interaction between QAPPT and Pt:

The interaction between the cationic QAPPT framework and Pt was examined by comparing the in-situ EC-SERS spectra and normal Raman spectrum of QAPPT. As shown in Figure S1a, the bands at 1287 and 1608 cm^{-1} in the normal Raman spectrum of a QAPPT membrane were found in the EC-SERS spectra, but those at 410, 787, 914 and 1211 cm^{-1} were absent. Figure S1b showed the details of the bands at 1287 and 1608 cm^{-1} at a narrow frequency region. First, these two bands were with identical frequency as that in the normal Raman spectrum of QAPPT and insensitive to potential shift. Usually, the SERS frequency of a species adsorbed on electrode surface was sensitive to potential shift, which is so-called vibrational Stark effect.[5-8] Second, these two bands were with identical bandwidth as that in the normal Raman spectrum of QAPPT. Third, there may be a vibrational signal between Pt and a chemically adsorbed molecule, which was absent in the EC-SERS spectra. These features indicated that the bands of 1287 and 1608 cm^{-1} in the SERS spectra were not from chemically adsorbed QAPPT on Pt surface but similar to that of QAPPT bulk. Therefore, they were attributed to the bulk QAPPT in the laser spot of a Raman spectrometer, and we excluded the chemical adsorption of QAPPT.

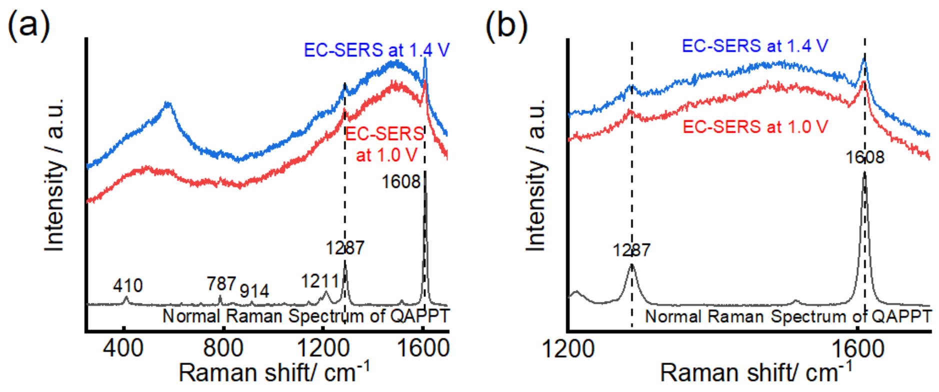

Figure S1. The normal Raman spectrum of QAPPT (black) and potential-dependent EC-SERS spectrum of Pt in QAPPT at 1.0 V (red) and 1.4 V (blue) within wide (a) and narrow (b) frequency regions.

S5 Molecular dynamics simulation:

All-atom molecular dynamics (MD) simulations were performed using the LAMMPS software package, with the CHARMM potential energy function employed to describe the QAPPT polymer and NaOH solution.[9, 10] The QAPPT polymer system consists of 1000 water molecules, 100 OH^- anions, and 10 QAPPT polymer chains, each containing 10 monomer units. In the NaOH model, to ensure complete solvation of Na^+ ions, the system includes 2 Na^+ ions, 2 OH^- ions, and 1000 water molecules. Both initial models were subjected to equilibration at 300 K and 1 bar for at least 10 ns, followed by NVE ensemble relaxation at 300 K for a minimum of 10 ns before structural analysis.

The QAPPT system has a simulation box size of 41.8 × 42.5 × 45 Å, while the NaOH system is contained within a box of dimensions 41.8 × 42.5 × 20 Å.

In molecular dynamics simulations, pre-equilibration procedures and initial structural optimization are essential for achieving thermodynamic equilibrium. Through long-time molecular dynamics (MD) simulations of the QAPPT system, a state of equilibrium was established. To rigorously validate the stability of the system, the radial distribution function (RDF) integration averaged over the trajectory frames sampled every 1 ns from the 10 ns simulations were analyzed to quantify the coordination number of H_2O molecules surrounding OH^- ions. As illustrated in Figure S1, the coordination numbers trended to 6.3 throughout all analyzed intervals, yielding an overall average coordination number of 6.3.

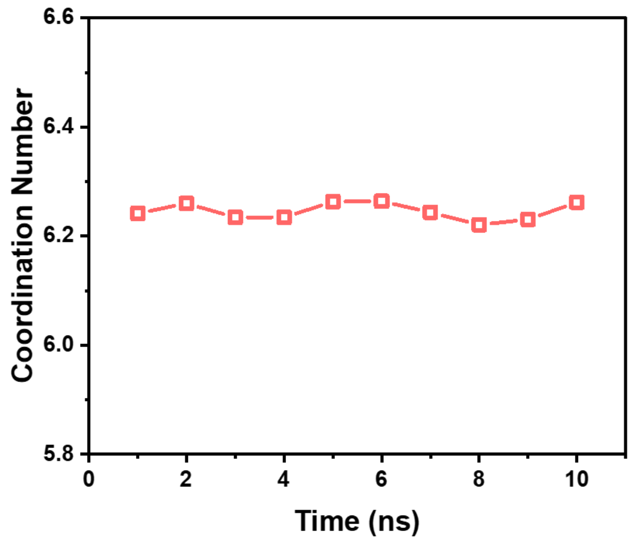

Figure S2. H_2O coordination numbers around OH^- during 10 ns simulations.

S6 Density functional theoretical calculation of the vibration of surface Pt hydroxides

Geometry optimization and vibrational frequency calculations of surface halides on Pt(111)-$\sqrt{3}\times\sqrt{3}$, Pt(553)-2×1, and Pt(533)-2×1 slabs were performed by using the DFT method in the Vienna ab initio simulation package.[11-14] The projector augmented wave method and the Perdew–Burke–Ernzerhof generalized gradient approximation functional were used.[15] An energy cutoff of 450 eV was applied. Monkhorst–Pack k-point sampling of 9 × 9 × 1 was applied to Pt(111), and that of 7 × 3 × 1 was applied to Pt(553) and Pt(533). The lattice constant was 3.9864 Å.[16] A vacuum layer with a thickness of 30 Å. In addition, to describe the species on the surface, two layers of Pt atoms were fixed at the bottom and the top three layers were allowed to relax.

S7 Quantum chemical calculation:

The $OH^-(H_2O)_n$ clusters were geometrically optimized by using MP2 method with a basis set of Aug-cc-PVTZ, whose energy included zero-point-energy correction. The single point energy of the optimized $OH^-(H_2O)_n$ clusters was further calculated at the level of CCSD/Aug-cc-PVQZ. The polarizable continuum model was employed to describe a solvation environment. All the calculation was carried out by using Gaussian 09 (version E.01).[17]

References

(1) Tian, N.; Zhou, Z.-Y.; Sun, S.-G.; Ding, Y.; Wang, Z. L., Synthesis of Tetrahexahedral Platinum Nanocrystals with High-Index Facets and High Electro-Oxidation Activity. *Science* **2007,** *316,* 732-735.

(2) Li, J.-F.; Yang, Z.-L.; Ren, B.; Liu, G.-K.; Fang, P.-P.; Jiang, Y.-X.; Wu, D.-Y.; Tian, Z.-Q., Surface-Enhanced Raman Spectroscopy Using Gold-Core Platinum-Shell Nanoparticle Film Electrodes: Toward a Versatile Vibrational Strategy for Electrochemical Interfaces. *Langmuir* **2006,** *22,* 10372-10379.

(3) Yuan, Q.; Wu, L.-W.; Huang, Y.-F., Making a Surface-Enhanced Raman Scattering-Active Pt Ultramicroelectrode by Electrochemically Adsorbing Au-core@Pt-shell Nanoparticles. *J. Phys. Chem. C* **2024,** *128,* 2022-2027.

(4) Wu, L.-W.; Huang, M.-L.; Yang, Y.-X.; Huang, Y.-F., *In-situ* electrochemical surface-enhanced Raman spectroscopy in metal/polyelectrolyte interfaces. *Chin. J. Catal.* **2022,** *43,* 2820-2825.

(5) Bishop, D. M., The vibrational Stark effect. *J. Chem. Phys.* **1993,** *98,* 3179-3184.

(6) Hush, N. S.; Reimers, J. R., Vibrational Stark Spectroscopy. 1. Basic Theory and Application to the CO Stretch. *J. Phys. Chem.* **1995,** *99,* 15798-15805.

(7) Lambert, D. K., Vibrational Stark effect of adsorbates at electrochemical interfaces. *Electrochim. Acta* **1996,** *41,* 623-630.

(8) Wasileski, S. A.; Koper, M. T. M.; Weaver, M. J., Field-Dependent Electrode–Chemisorbate Bonding: Sensitivity of Vibrational Stark Effect and Binding Energetics to Nature of Surface Coordination. *J. Am. Chem. Soc.* **2002,** *124,* 2796-2805.

(9) Thompson, A. P.; Aktulga, H. M.; Berger, R.; Bolintineanu, D. S.; Brown, W. M.; Crozier, P. S.; in 't Veld, P. J.; Kohlmeyer, A.; Moore, S. G.; Nguyen, T. D. et al., LAMMPS - a flexible simulation tool for particle-based materials modeling at the atomic, meso, and continuum scales. *Comput. Phys. Commun.* **2022,** *271,* 108171.

(10) MacKerell, A. D., Jr.; Bashford, D.; Bellott, M.; Dunbrack, R. L., Jr.; Evanseck, J. D.; Field, M. J.; Fischer, S.; Gao, J.; Guo, H.; Ha, S. et al., All-Atom Empirical Potential for Molecular Modeling and Dynamics Studies of Proteins. *J. Phys. Chem. B* **1998,** *102,* 3586-3616.

(11) Kresse, G.; Hafner, J., *Ab initio* molecular dynamics for liquid metals. *Phys. Rev. B* **1993,** *47,* 558-561.

(12) Kresse, G.; Hafner, J., *Ab initio* molecular-dynamics simulation of the liquid-metal-amorphous-semiconductor transition in germanium. *Phys. Rev. B* **1994,** *49,* 14251-14269.

(13) Kresse, G.; Furthmuller, J., Efficient iterative schemes for ab initio total-energy calculations using a plane-wave basis set. *Phys. Rev. B* **1996,** *54,* 11169-11186.

(14) Kresse, G.; Furthmuller, J., Efficiency of ab-initio total energy calculations for metals and semiconductors using a plane-wave basis set. *Comput. Mater. Sci.* **1996,** *6*, 15-50.

(15) Perdew, J. P.; Burke, K.; Ernzerhof, M., Generalized Gradient Approximation Made Simple. *Phys. Rev. Lett.* **1996,** *77*, 3865-3868.

(16) Kolb, M. J.; Calle-Vallejo, F.; Juurlink, L. B. F.; Koper, M. T. M., Density functional theory study of adsorption of H_2O, H, O, and OH on stepped platinum surfaces. *J. Chem. Phys.* **2014,** *140*.

(17) Frisch, M. J.; Trucks, G. W.; Schlegel, H. B.; Scuseria, G. E.; Robb, M. A.; Cheeseman, J. R.; Scalmani, G.; Barone, V.; Mennucci, B.; Petersson, G. A. et al. *Gaussian*, E.01; Gaussian, Inc.: Wallingford CT, 2009.
